# Physics-informed hybrid reinforcement learning for estimating lithium-ion battery state of health

**DOI:** 10.1038/s41598-025-30602-4

**Published:** 2025-12-15

**Authors:** Nermin M. Salem, Ahmed Mohamed

**Affiliations:** 1 Electrical Engineering Department, Faculty of Engineering and Technology, Future University in Egypt (FUE), Cairo, 11835 Egypt; 2 Electrical Engineering Department, Grove School of Engineering, City College of New York, New York, NY 10031 USA

**Keywords:** Battery degradation, Battery health estimation, Battery management systems (BMS), Data-driven prediction, Deep reinforcement learning, Hybrid modeling, Lithium-ion batteries (LIB), LSTM, Physics-informed reward function, PPO, RL, SOH, Time series forecasting, Energy science and technology, Engineering, Mathematics and computing

## Abstract

Although Data-driven methods are becoming widely applied for estimating the state of health (SOH) of lithium-ion batteries, they often suffer from a lack of interpretability and generalization capabilities. To address these limitations, this research proposes a hybrid methodology that combines Long Short-Term Memory (LSTM) networks and Reinforcement Learning (RL) using Proximal Policy Optimization (PPO) to enhance both SOH prediction accuracy and model interpretability. The proposed methodology begins by training an LSTM model on key battery features for two different operational profiles, thereby capturing the temporal dependencies present in battery degradation. To improve interpretability and adaptive learning, a hybrid model is then introduced, where a PPO agent corrects the LSTM predictions based on a reward function designed to account for physical and monotonic degradation constraints of the SOH. Additionally, two models are trained separately for comparison: a pure RL-based model and an LSTM-based model, which enables comparison between data-driven and control-driven learning. The models are initially trained and validated on the NASA battery dataset and further evaluated on a separate real-world dataset of CALCE CS2 battery cells to assess robustness. Experimental results demonstrated that the hybrid model significantly enhances the robustness and accuracy of SOH prediction, outperforming both standalone LSTM and RL models. Statistical metrics, such as Mean Absolute Error (MAE) and Root Mean Square Error (RMSE), were reduced while ${R}^{2}$ score has increased significantly. At the same time, interpretable results are improved through an RL feedback mechanism grounded in physical behavior.

## Introduction

Lithium-Ion Batteries (LIBs) are widely deployed across various domains, including electric vehicles (EVs), renewable energy storage, aerospace systems, and portable electronics, owing to their high energy density, low self-discharge rate, and long cycle life^1–3^. A critical factor in ensuring the reliability, safety, and longevity of these batteries is accurate estimation of their SOH, which quantifies the remaining usable capacity relative to the battery’s original performance.

In recent years, data-driven approaches^4^ have gained popularity for SOH estimation due to their ability to model complex, nonlinear degradation behaviors without requiring explicit knowledge of internal electrochemical processes. Among these, LSTM^5^ networks have shown promise in capturing temporal dependencies and long-term trends in time series battery data. However, while LSTMs can achieve high predictive performance, they often act as black-box models, lacking transparency and physical consistency, two essential traits for real-world deployment in safety–critical systems.

To address the shortcomings of pure data-driven models, this paper proposes a hybrid modeling framework that integrates LSTM with RL, specifically PPO. In this framework, the LSTM serves as a base initial predictor trained on battery operational data, including voltage, current, temperature, and cycle index data. A PPO agent is then trained to adjust the LSTM’s predictions through an adaptive correction mechanism. The RL agent interacts with a custom-designed environment, where rewards are shaped by both prediction accuracy and physically motivated constraints, such as the expected monotonic degradation of SOH.

Therefore, the incorporation of Reinforcement Learning (RL) in our hybrid framework serves two primary purposes.

1. RL enhances the adaptability of the model by dynamically correcting LSTM predictions based on real-time feedback through the reward mechanism. This enables the model to handle varying degradation behaviors under different operating conditions more effectively.
2. RL improves physical interpretability by embedding domain-specific constraints, such as the monotonic degradation trend of SOH, into the reward function. As a result, the RL component guides the model toward physically consistent predictions, thereby overcoming a key limitation of purely data-driven approaches, such as standalone LSTM models.

Physics-Informed Machine Learning (PIML) is an emerging paradigm that integrates domain-specific physical laws and constraints into data-driven models to enhance their generalization, interpretability, and reliability. By embedding governing equations, monotonicity conditions, or conservation principles into the training process, PIML bridges the gap between purely empirical modeling and first-principles simulation. In the context of battery health prediction, PIML ensures that model outputs adhere to known degradation behaviors, such as the irreversible decline of capacity over time, thereby preventing physically implausible predictions^2,6,7^. This approach not only improves predictive robustness in scenarios with limited or noisy data but also enhances trust in model decisions, making it particularly suitable for safety–critical applications, such as BMS. In this work, PIML principles are incorporated into the reinforcement learning framework through a reward function that penalizes violations of expected SOH degradation trends, aligning the model’s learning trajectory with physical reality.

The key contributions of this work are as follows:

1. A novel LSTM + PPO hybrid architecture for battery SOH prediction, which combines temporal feature extraction with constraint-aware correction.
2. A custom reward function that embeds domain knowledge into the learning process by penalizing violations of physical degradation behavior.
3. A comparative evaluation of the hybrid model against standalone LSTM and RL-only models on NASA’s battery dataset^8^, demonstrating improved predictive accuracy and physical interpretability.
4. To further evaluate the effectiveness and generalization of our model, the CALCE CS2 dataset^9^ was used.

This hybrid framework bridges the gap between the predictive power of deep learning and the interpretability of physics-informed modeling, offering a robust and extensible solution for real-time battery health diagnostics.

The rest of this paper is organized as follows. “Literature review” section introduces the literature review. “Research methodology” section provides research methodology. “Experiments and analysis” section provided a detailed overview of the experiments and analysis. “Evaluation on CALCE CS2 dataset” section provided an evaluation of our model on the CALCE CS2 dataset. Conclusions are summarized in “Conclusion” section.

## Literature review

Accurate estimation of battery SOH is crucial for ensuring the reliability and safety of energy storage systems, particularly in applications such as electric vehicles and grid-scale storage. Generally, there are two methods for estimating the SOH of a lithium-ion battery. Traditional methods for SOH estimation include electrochemical modeling, such as equivalent circuit models (ECM) and physics-based models, which rely on detailed internal parameters^10^. Although these methods offer interpretability and physical consistency, they often require extensive system identification and are computationally expensive. Data-driven approaches have gained popularity for SOH prediction with the growing availability of battery operational data. Machine learning (ML) models such as Support Vector Regression (SVR), Random Forests, and more recently, Deep Learning (DL) architectures like Convolutional Neural Networks (CNNs) and LSTM networks have shown strong predictive performance^11,12^. LSTM networks are effective at capturing temporal dependencies in degradation patterns; however, they often function as black-box models, which limits their interpretability in safety–critical contexts. A recent novel study^13^ proposed a hybrid SOH prediction framework based on Variational Mode Decomposition–Permutation Entropy–Improved Dung Beetle Optimization–Temporal Convolutional Network (VMD–PE–IDBO–TCN). In this method, the VMD–PE process decomposes the non-stationary battery degradation signals into several stationary subsequences, thereby mitigating noise and nonlinearity in the raw capacity data. Each subsequence is modeled using a Temporal Convolutional Network (TCN), whose parameters are fine-tuned using an Improved Dung Beetle Optimization (IDBO) algorithm that integrates chaotic mapping, a golden sine strategy, and adaptive Gaussian–Cauchy mutation to enhance global search and convergence. The reconstructed predictions from all subsequences are combined to yield the final SOH estimate.

To address this challenge, recent research has explored PIML techniques, which incorporate physical constraints or domain knowledge directly into the learning process^6^. A foundational example is the Physics-Informed Neural Network (PINN) framework introduced by Raissi et al.^14^, which embeds differential equations into the loss function. In battery applications, PIML methods have been employed to constrain SOH predictions by utilizing electrochemical degradation laws, monotonic capacity fade rules, or thermodynamic limits^15^. These hybrid approaches help bridge the gap between empirical performance and physical realism, improving both robustness and trustworthiness of the models. Wang et al.^16^ employed a Physics-Informed Neural Network (PINN) framework for robust SOH estimation across various battery chemistries. While the model demonstrated strong generalization capabilities, its performance remained sensitive to measuring noise and fluctuating operating conditions. To address cross-domain adaptability, Vilsen et al.^17^ proposed a physics-guided transfer learning approach that enhanced model generalization across different battery types. Nonetheless, noticeable performance degradation was observed during domain transitions. Ye et al.^18^ proposed a Physics-Informed Feedforward Neural Network (PIFNN) for lithium-ion battery SOH estimation, designed to improve both accuracy and interpretability. The method extracts features from incremental capacity (IC) and differential temperature curves, with IC peaks reflecting electrochemical reactions linked to material degradation. By converting the monotonic relationship between IC peaks and SOH into physical constraints, the model incorporates domain knowledge during learning. A secondary physics-constrained training step is then applied to refine predictions, ensuring they align with known battery aging mechanisms. Sun et al.^19^, proposed a method called Battery Physics-Informed Neural Network (BPINN), to enhance the interpretability of the Feedforward Neural Network (FNN) in SOH prediction. This approach constrains the model’s learning process, ensuring that its predictions align more closely with physical reality. To evaluate the effectiveness of integrating physical constraints and subsequent optimization in the BPINN model, two PIFNN variants were designed. BPINN-1 represents a version with only physical constraint integration, but no subsequent optimization. In contrast, BPINN-2 represents complete implementation, integrating physical constraints and subsequently optimizing it, for comparison with the traditional FNN.

Another promising direction is the integration of RL^20^ with data-driven predictors. RL has been employed for battery charging optimization and health estimation, where agents learn decision policies via interaction with an environment^21^. However, standalone RL methods often struggle with convergence and data efficiency in continuous, high-dimensional spaces.

To address these limitations, PINNs and Physics-guided reinforcement learning (RL) have embedded domain knowledge into data-driven models by constraining learning with physical principles. While PINNs incorporate governing equations directly into the optimization process, they require explicit mathematical models of the underlying dynamics, which are often unavailable or difficult to derive for battery degradation. Physics-guided RL approaches, on the other hand, typically impose constraints during training but often operate in an end-to-end manner without separating temporal feature extraction from constraint enforcement. In contrast, our work introduces a two-stage hybrid design, where an LSTM first captures temporal degradation patterns and a PPO agent subsequently corrects predictions using a custom reward function that enforces monotonic degradation behavior. This design enables improved interpretability, flexibility when explicit physics is unavailable, and superior predictive performance compared to both standalone LSTM and RL models.

## Research methodology

**Fig. 1:**
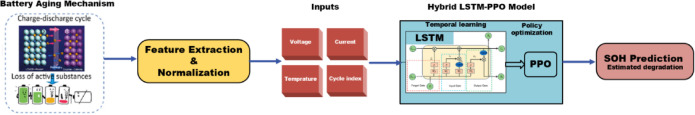
Hybrid model overview.

This study proposes a hybrid LSTM + PPO approach, as illustrated in Fig. 1, which presents an overview of the proposed hybrid framework for SOH estimation. Raw charge–discharge data are processed into voltage, current, temperature, and cycle index features. The LSTM captures temporal dependencies, while the PPO refines predictions through policy optimization to estimate SOH degradation. Experimental measurements from the NASA battery dataset (voltage, current, temperature, and cycle index) are used to construct features that are input into the LSTM to capture temporal dependencies in degradation. The LSTM predictions are then refined by a PPO agent, which applies constraint-aware corrections guided by a custom reward function that enforces monotonic degradation behavior. The final output is a SOH estimation that strikes a balance between predictive accuracy and physical interpretability.

The model is trained and evaluated using real-world battery degradation datasets from the publicly available battery dataset provided by NASA^8^. The dataset includes complete lifecycle data for four different models of 18,650 lithium-ion batteries (B0005, B0006, B0007, and B0018) under various operating conditions. Different specifications of batteries were considered in this research, where batteries B0005, B0006, and B0007 utilize lithium nickel cobalt aluminum oxide (NCA) as the positive electrode and graphite as the negative electrode, with a nominal capacity of 2 Ah. At the same time, battery B0018 utilizes lithium nickel manganese cobalt oxide (NMC) for the positive electrode and graphite for the negative electrode, with a nominal capacity of 1.35 Ah. All batteries underwent charging using a constant current-constant voltage (CC-CV) protocol at 1.5 A, with a cutoff voltage of 4.2 V and a cutoff current of 20 mA. Discharge conditions varied: B0005, B0006, and B0007 were discharged at 2 A with cutoff voltages of 2.7 V, 2.5 V, and 2.2 V, respectively, while B0018 was discharged at 1.5 A down to 2.5 V^19^. All tests were performed at a room temperature (24 °C). The dataset originally included measurements of battery Voltage, Current, temperature, Capacity, and time. The SOH for each cycle is computed as:

1 $${SOH}_{n}=\frac{{C}_{n}}{{C}_{0}}$$

where, ${C}_{n}$ is the measured capacity of the battery at ${n}^{th}$ cycle and ${C}_{0}$ is the initial capacity, typically taken as the average capacity during the first full cycle. This normalization ensures that all batteries, regardless of their nominal capacity or charge protocol, have a starting SOH of approximately 1.0, enabling a direct comparison of degradation patterns across different units. Using this method of SOH calculation is considered an intuitive and straightforward approach, as it is both simple and provides a generalizable method applicable across different battery chemistries and manufacturers. Additionally, it yields a continuous, bounded scalar value, making it ideal for regression tasks in deep learning and reinforcement learning methods.

**Fig. 2:**
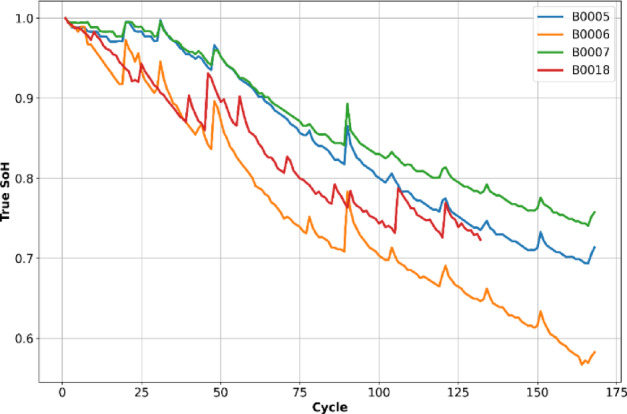
SOH over battery life.

**Fig. 3:**
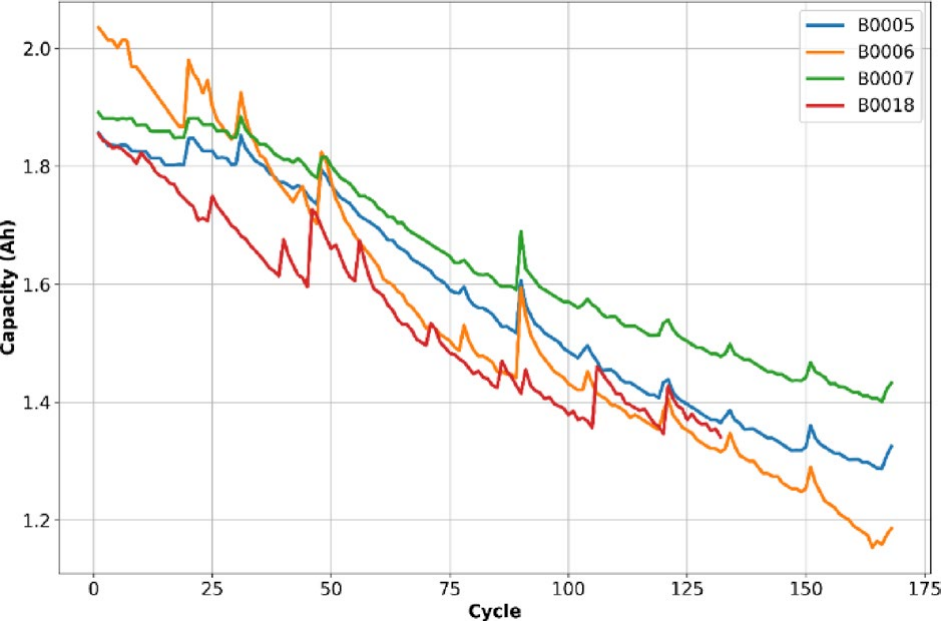
Battery capacity over charge–discharge cycles.

Figures 2 and 3 illustrate the SOH and capacity degradation curves of the four different batteries of the NASA dataset as a function of cycles. As shown, the capacity of each battery decreases with the increase in the number of cycles. The four batteries were selected based on their completeness and the variety of degradation profiles they exhibit. Each dataset comprises multiple charge/discharge cycles recorded under varying operating conditions. To enable effective time-series modeling, each battery’s data is processed for the following parameters: Voltage, Current, Temperature, and Cycle index. These parameters were chosen because they effectively capture the electrochemical, thermal, and aging characteristics of lithium-ion batteries and are readily available from BMS. Capacity was not used as an input variable, as it serves as the reference quantity for computing the SOH and thus forms the target output of the model rather than an input feature. Table 1 summarizes input features and their physical significance in SOH estimation.

**Table 1:** The input features’ contribution to SOH estimation.

Feature	Physical significance	Role in SOH estimation
Voltage	Reflects state of charge (SOC), internal resistance, and degradation behavior	Captures electrochemical wear and potential shifts due to aging
Current	Indicates applied load and electrochemical stress during cycling	Accounts for discharge-induced variations and stress effects
Temperature	Influences the aging rate, SEI growth, and reaction kinetics	Models thermal degradation effects and potential runaway risks
Cycle Index	Represents the number of completed charge–discharge cycles	Serves as a temporal indicator of cumulative battery aging

For each battery, raw time-series data is grouped by cycle, and all features are normalized using MinMaxScaler to values [0, 1]. The aggregated and normalized cycle-level data is then used to construct fixed-length input sequences; a sliding window of size 10, suitable for LSTM training. Sliding windows of fixed length are created to generate input sequences for the LSTM model. Each sequence is used to predict the SOH at the subsequent cycle. This process yields a supervised dataset of sequential input features and corresponding SOH targets, facilitating temporal learning. This process yields a supervised dataset of sequential input features and corresponding SOH targets, facilitating temporal learning.

To accurately model and predict the battery’s SOH over its lifecycle, this study employs a hybrid machine learning framework that combines an LSTM network for temporal sequence modeling and PPO from deep reinforcement learning (DRL) for adaptive prediction refinement. This hybrid design leverages the strength of supervised learning to capture long-term degradation trends and reinforcement learning to fine-tune predictions in a self-correcting manner.

LSTM is a type of recurrent neural network (RNN) specifically designed to learn and retain long-term dependencies in sequential data^22^. Unlike traditional RNNs, which suffer from vanishing or exploding gradients during training, LSTM networks^23,24^ incorporate gating mechanisms, namely the input, forget, and output gates, which regulate the flow of information through the cell.

These gates enable LSTM units to selectively retain relevant features over long time spans, making them particularly effective for modeling time-series data, such as battery aging.

The operations inside an LSTM cell at time step *t* are computed by:

$${f}_{t}=\upsigma \backslash big\left({W}_{f}\cdot \left[{h}_{t-1},{x}_{t}\right]+{b}_{f}\backslash big\right)$$

$${i}_{t}=\upsigma \backslash big\left({W}_{i}\cdot \left[{h}_{t-1},{x}_{t}\right]+{b}_{i}\backslash big\right)$$

$$\widetilde{{C}_{t}}=\text{tanh}\backslash big\left({W}_{C}\cdot \left[{h}_{t-1},{x}_{t}\right]+{b}_{C}\backslash big\right)$$

$$C_{t} = f_{t} \odot C_{t - 1} + i_{t} \odot \tilde{C}_{t}$$

$${o}_{t}=\upsigma \backslash big\left({W}_{o}\cdot \left[{h}_{t-1},{x}_{t}\right]+{b}_{o}\backslash big\right)$$

2 $${h}_{t}={o}_{t}\odot \text{tanh}\left({C}_{t}\right)$$

where, ${x}_{t}$ is the input vector at time *t,* ${h}_{t-1}$ is the previous hidden state, ${f}_{t}$, ${i}_{t}{ o}_{t}$ are the forget, input and out gates, respectively, ${C}_{t}$ is the cell state, $\sigma (.)$ denotes the sigmoid activation and $\odot$ is the element-wise multiplication.

Therefore, the LSTM model is implemented to capture the temporal dependencies in battery degradation. The model architecture consists of:

- Input layer shaped according to sequence length and feature dimensions
- Two stacked LSTM layers with 128 and 64 units, respectively
- A dropout layer with a dropout rate of 0.3 for regularization
- A fully connected dense layer with ReLU activation
- An output layer producing a scalar SOH prediction

Our LSTM model is compiled using the Huber loss function^25^, computed as:

3 $${L}_{\delta }\left(a\right)= \left\{\begin{array}{c}\frac{1}{2}{a}^{2} for \left|a\right|\le \delta \\ \delta (\left|a\right|-\frac{1}{2}\delta ) otherwise\end{array}\right.$$

where, $a = {y}_{true}-{y}_{pred}$ and $\delta$ is the threshold hyperparameter.

This loss formula balances robustness to outliers and smooth optimization. The Adam optimizer is used for training. Early stopping is applied to avoid overfitting based on validation loss. Table 2 shows the LSTM Model Layout for Battery B0018 Data. The LSTM model serves as the guideline for the Reinforcement Learning-Based Refinement with PPO, acting as a baseline model.

**Table 2:** LSTM model layout for battery B0018 data.

Layer	Output shape	Param #
lstm (LSTM)	(None, 10, 128)	68,096
dropout (Dropout)	(None, 10, 128)	0
lstm_1 (LSTM)	(None, 64)	49,408
dense (Dense)	(None, 32)	2,080
dense_1 (Dense)	(None, 1)	33
Totals: • Total params: 119,617 (467.25 KB) • Trainable params: 119,617 (467.25 KB) • Non-trainable params: 0 (0.00 B)		

To address the limitations of static sequence modeling and introduce adaptability, RL^26^ is employed to learn a policy for SOH prediction using direct reward feedback. The environment is modeled as a Markov Decision Process (MDP) defined by the tuple $(S, A, P, R, \gamma )$, where $S$ is the state space, normalized battery features, $A$ is the action space, in the proposed hybrid algorithm, is the correction term for the initial predicted SOH, $P$ is the transition probability function, deterministic per time step, $R$ is the reward function and $\gamma$ is the discount factor.

PPO training stability and sample efficiency by limiting the extent to which the policy can change at each update step. It achieves this through a clipped surrogate objective function, preventing significant policy updates that could destabilize learning.

The PPO agent acts as a correction mechanism. The LSTM generates a preliminary SOH prediction, and the PPO agent learns an additive correction to refine this prediction. This hybridization enables the model to leverage the temporal understanding of the LSTM while incorporating the flexibility of RL to adaptively respond to prediction errors, noise, and unmodeled dynamics. The hybridization improves generalization by merging inductive biases from both domains. The hybrid prediction at time step *t* is computed from:

4 $${\widehat{y}}_{t}^{hybrid}={\widehat{\text{y}}}_{t}^{LSTM}+{\Delta }_{t}$$

where, ${\widehat{\text{y}}}_{t}^{LSTM}$ is the output of the pretrained LSTM model and ${\Delta }_{t}$ is the correction term generated by the PPO agent.

This correction term is modeled as ${a}_{t}\in R$ selected by a stochastic policy ${\pi }_{\theta }\left({a}_{t}|{s}_{t}\right)$, conditioned on the current state vector ${s}_{t}\in {R}^{n}$, which contains normalized battery measurements (voltage, current, temperature, cycle index). The PPO agent^27^ optimizes a clipped surrogate objective expressed in (5) to prevent significant policy updates and ensure stable learning:

5 $${L}^{\text{CLIP}}\left(\uptheta \right)={E}_{t}\left[\text{min}\left({r}_{t}\left(\uptheta \right)\widehat{{A}_{t}},{\text{clip}}\left({r}_{t}\left(\uptheta \right),1-\upepsilon ,1+\upepsilon \right)\widehat{{A}_{t}}\right)\right]$$

where, ${r}_{t}\left(\uptheta \right)=\frac{{\uppi }_{\uptheta }\left({a}_{t}|{s}_{t}\right)}{{\uppi }_{{\uptheta }_{\text{old}}}\left({a}_{t}|{s}_{t}\right)}$ is the probability ratio, $\widehat{{A}_{t}}$ is the estimated advantage function and $\epsilon$ is the clipping threshold. The default value in stable_baseline3. PPO^28^ is used,$\epsilon = 0.2$. This controls how far the new policy is allowed to deviate from the old policy during training.

For the PPO Model, a custom OpenAI Gym environment is developed where the state $S$ had the current normalized cycle features, action $A$ represents an additive correction to the LSTM output, and a reward function defined to penalize significant prediction errors and non-monotonic increases in SOH, encouraging physical plausibility. The reward ${r}_{t}$ at battery cycle $n$ is computed:

6 $${r}_{n}= -\left|{\widehat{y}}_{n}-{y}_{n}\right|-0.2 \mathit{max}{(0, \widehat{y}-{\widehat{y}}_{n-1})}^{2}+0.05(1-\left|\widehat{y}-y\right|)$$

where, ${\widehat{y}}_{n}$ is the predicted SOH for cycle $n$, ${y}_{n}$ is the true SOH at cycle $n$ and ${\widehat{y}}_{n-1}$ is the predicted SOH at the previous cycle $n-1$. The reward function designed in the proposed hybrid PPO model can be interpreted as physics-informed because it embeds physical knowledge and constraints about battery degradation and health into the learning process.

The reward function is composed of three components. The first component is the absolute error penalty, the primary component, which penalizes the agent based on the absolute error between its prediction and the ground truth. It directly incentivizes accurate SOH predictions. It serves as a loss-like penalty, guiding the RL agent toward minimizing prediction errors.

The second component is the monotonicity penalty, which penalizes non-physical increases in predicted SOH. Since battery health degrades over time, a significant upward jump in predicted SOH is deemed implausible. The quadratic form ensures more substantial penalties for larger violations, while predictions that follow a monotonic (decreasing or constant) trend incur no penalty. This term aligns with real-world battery degradation dynamics, encouraging the agent to respect domain-specific physical constraints.

The third component in the proximity bonus, this positive reinforcement term rewards predictions that are very close to the true SOH. Although small in magnitude compared to the error penalty, this term encourages the agent to fine-tune its predictions even after achieving general accuracy. A small bonus for high-accuracy predictions nudges the model toward precision once general accuracy is achieved. The structure of the reward function is carefully designed to strike a balance between exploration and physical validity. This formulation enables the RL agent to learn both data-driven patterns and physically consistent behavior, a critical requirement for real-world battery health monitoring systems.

The weights of the reward function were empirically chosen after exploratory experiments to balance the prediction loss against monotonicity penalties. These values ensured that physical constraints influenced learning without overwhelming predictive accuracy. These values ensured that physical constraints influenced learning without overwhelming predictive accuracy to balance three objectives:

- Minimizing prediction error with respect to true SOH
- Discouraging non-physical increases in SOH (monotonic degradation constraint)
- Rewarding predictions that remain close to the actual trajectory. These weights were tuned through preliminary experiments to stabilize training and avoid over-penalization of correction actions.

To summarize, the training process works as follows:

- Pretrain LSTM: The LSTM model is first trained on a supervised regression task using historical battery data. This captures coarse SOH degradation dynamics.
- Freeze LSTM: During PPO training, the LSTM weights remain frozen to ensure stable feature extraction.
- Train PPO Agent: The PPO agent is trained in an environment where each state consists of the current normalized sensor features, and the reward is computed based on the hybrid prediction’s accuracy and smoothness.

## Experiments and analysis

To evaluate the performance and generalization of the proposed hybrid methodology, we compare it with two other models: the LSTM-only and RL-only models. All model training and evaluations were conducted on Google Colab using a Tesla T4 GPU. The T4 provides 16 GB of GDDR6 memory and is optimized for deep learning inference and training tasks. The experiments utilized Python 3.10 with TensorFlow 2.x, Keras, scikit-learn, and Stable-Baselines3^28^ for reinforcement learning implementations. The LSTM model was trained for up to 250 epochs with an early stopping criterion (patience = 5) and a batch size of 16. For reinforcement learning, the PPO-based hybrid and RL-only agents were trained over 1,000,000 timesteps using default hyperparameters, including a clipping threshold (clip_range) of 0.2. All experiments were repeated using consistent seeds to ensure reproducibility.

Three evaluation metrics were standard regression metrics: MAE, RMSE, and the coefficient of determination (R^2^ score). MAE provides a direct measure of the average magnitude of prediction errors, indicating how close the predicted values are to the actual SOH without considering their direction. RMSE, on the other hand, emphasizes larger errors by squaring the residuals before averaging, making it more sensitive to outliers in the prediction. Lastly, the R^2^ score quantifies how well the model explains the variance in the actual SOH values, with a score closer to 1 indicating better predictive performance. Together, these metrics provide a comprehensive evaluation of the model’s accuracy, robustness, and reliability throughout the entire battery life cycle. These metrics are computed as follows:

7 $${\text{MAE}}=\frac{1}{{\varvec{N}}}{\sum }_{{\varvec{i}}=1}^{{\varvec{N}}}\left|\widehat{{{\varvec{y}}}_{{\varvec{i}}}}-{{\varvec{y}}}_{{\varvec{i}}}\right|$$

8 $${\text{RMSE}}=\sqrt{\frac{1}{{\varvec{N}}}{\sum }_{{\varvec{i}}=1}^{{\varvec{N}}}{\left(\widehat{{{\varvec{y}}}_{{\varvec{i}}}}-{{\varvec{y}}}_{{\varvec{i}}}\right)}^{2}}$$

9 $${{\varvec{R}}}^{2}\boldsymbol{ }=\boldsymbol{ }1-\frac{{\sum }_{{\varvec{i}}}{\left(\widehat{{{\varvec{y}}}_{{\varvec{i}}}}-{{\varvec{y}}}_{{\varvec{i}}}\right)}^{2}}{{\sum }_{{\varvec{i}}}{\left({{\varvec{y}}}_{{\varvec{i}}}-\overline{{\varvec{y}} }\right)}^{2}}$$

where, *N* is the number of samples to be evaluated, ${{\varvec{y}}}_{{\varvec{i}}}$ and $\widehat{{{\varvec{y}}}_{{\varvec{i}}}}$ are the actual and predicted SOH values, respectively.

The LSTM model effectively captures temporal dependencies in the battery degradation sequence, yielding relatively strong performance with low MAE and RMSE values, as well as a high R^2^ score. However, it lacks adaptive correction capabilities during inference, making it vulnerable to accumulated prediction errors, especially in later cycles.

The RL-only model using PPO learns to directly predict SOH based solely on features without leveraging any prior learned temporal structure. The PPO agent directly learns to predict SOH values at each cycle, using features from the current cycle’s data as input. The reward function penalizes deviation from the true SOH, non-monotonic behavior, and rewards closeness to the actual value. Over time, the agent learns a policy that maps raw input features to optimal SOH predictions through interaction with the environment. The SOH predicted for the RL-only is computed as:

10 $${\widehat{{\varvec{y}}}}_{{\varvec{t}}}^{{\varvec{R}}{\varvec{L}}}=\boldsymbol{ }{{\varvec{a}}}_{{\varvec{t}}},\boldsymbol{ }{{\varvec{a}}}_{{\varvec{t}}}\boldsymbol{ }\sim {{\varvec{\pi}}}_{{\varvec{\theta}}}\left({{\varvec{a}}}_{{\varvec{t}}}|{{\varvec{s}}}_{{\varvec{t}}}\right)$$

where, ${\widehat{{\varvec{y}}}}_{{\varvec{t}}}^{{\varvec{R}}{\varvec{L}}}$ is the predicted SOH by the RL model at time ${\varvec{t}}$, ${{\varvec{a}}}_{{\varvec{t}}}$ is the action output of the RL policy, i.e., equal to the predicted SOH, ${{\varvec{\pi}}}_{{\varvec{\theta}}}\left({{\varvec{a}}}_{{\varvec{t}}}|{{\varvec{s}}}_{{\varvec{t}}}\right)$ is the stochastic policy, parameterized by ${\varvec{\theta}}$, which samples the action based on the current state ${{\varvec{s}}}_{{\varvec{t}}}$.

While it demonstrates some adaptability due to its reward-based training, its performance generally lags that of the LSTM and hybrid methods in terms of accuracy. It often exhibits higher MAE and RMSE values, along with a lower R^2^ score, indicating less reliable and noisier predictions.

All models were trained and tested on the NASA dataset^8^. For each battery cell, the initial portion of the cycle life was used for training, and the remaining portion for testing. Specifically, 60% of the total cycles (starting from Cycle 1) were used for training, while the remaining 40% were used for testing and validation. This split ensures that the model learns degradation patterns from the early-to-mid-life region and is evaluated on unseen later-life data, reflecting real-world prediction scenarios. To evaluate the performance and generalization, and for a broader perspective of our hybrid LSTM + PPO, we also include results reported in recent physics-informed approaches, specifically physics-informed machine learning. We compared it with BPINN-1, BPINN-2, and FNN models reported in^19^.

**Fig. 4:**
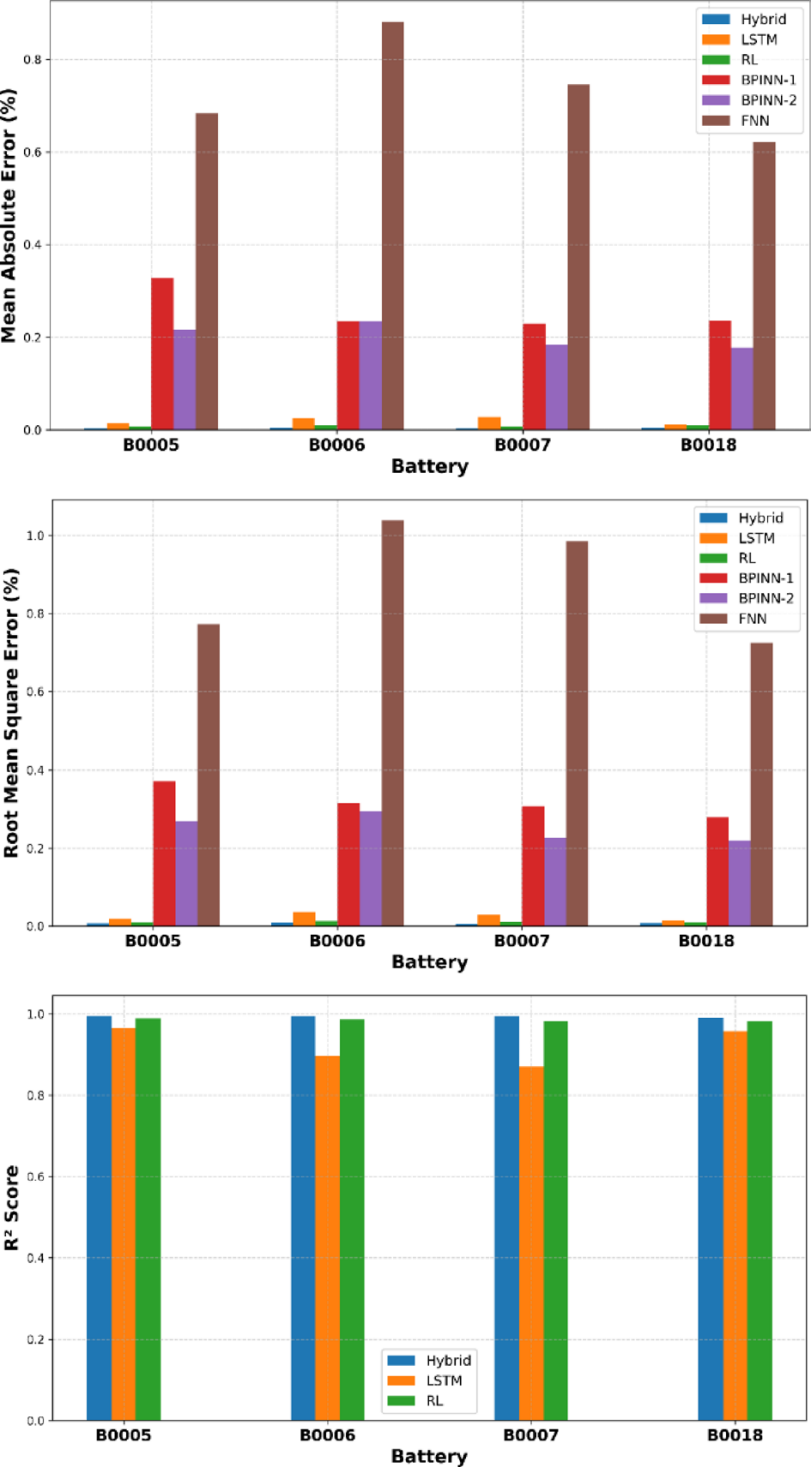
Evaluation metrics comparison for different NASA batteries.

**Fig. 5:**
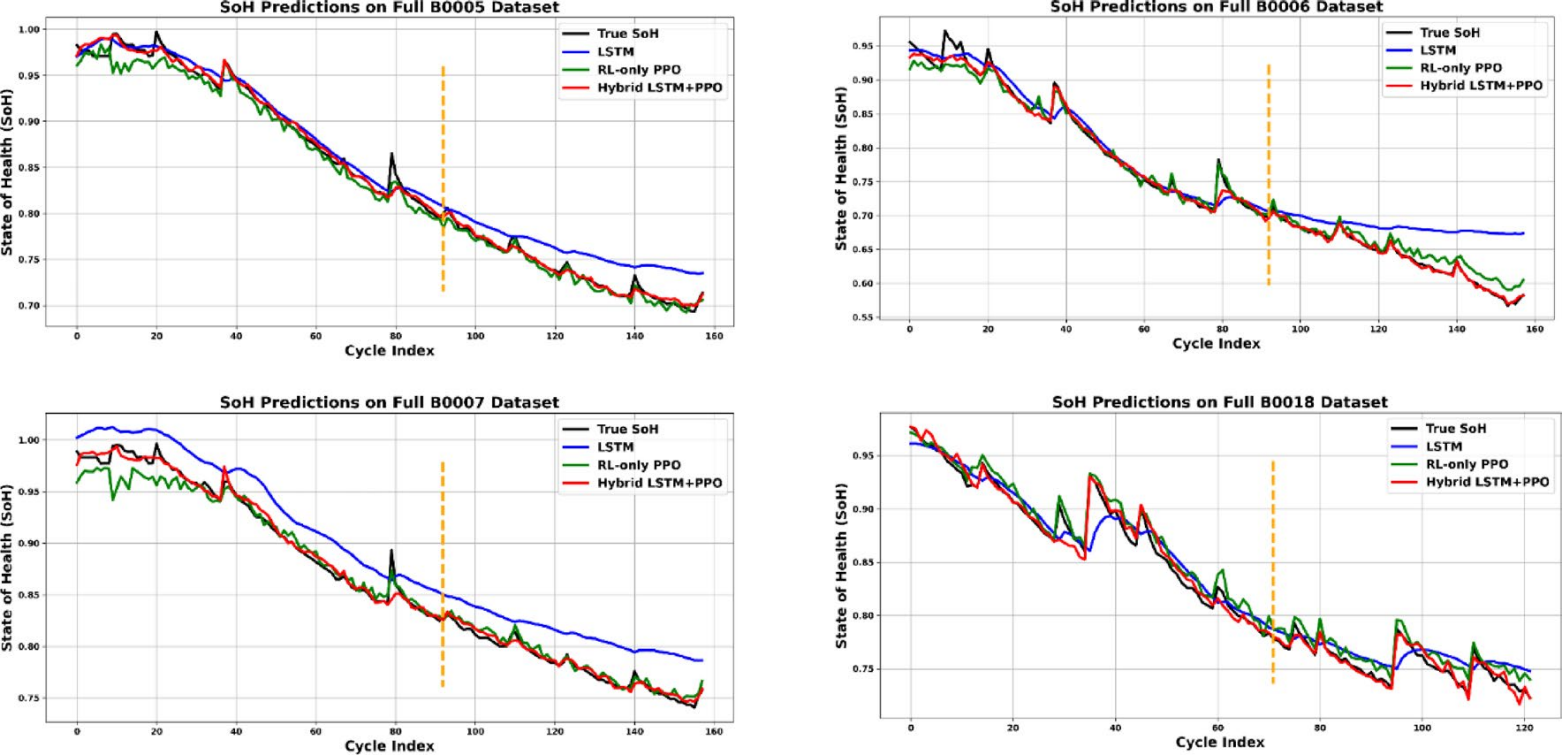
SOH Prediction of the NASA Batteries for the Proposed Hybrid LSTM + PPO, LSTM-Only, and RL-only PPO

A comprehensive comparative analysis of the experimental outcomes is summarized numerically in Table 3 and illustrated in Fig. 4. Fig. 5 demonstrates the predictions of the whole life cycle across different batteries of the NASA dataset.These results clearly highlight the superior performance of the proposed approach compared to conventional methods, both visually and quantitatively.

**Table 3:** Evaluation metrics of the NASA dataset batteries.

Battery		B0005	B0006	B0007	B0018
MAE (%)	**Hybrid**	**0.0038**	**0.0050**	**0.0039**	**0.0048**
	LSTM	0.0147	0.0253	0.0283	0.0121
	RL	0.0072	0.0093	0.0072	0.0092
	BPINN-1^19^	0.3274	0.2351	0.2294	0.2365
	BPINN-2 ^19^	0.2164	0.2351	0.1837	0.1771
	FNN^19^	0.6826	0.8813	0.7453	0.6216
RMSE (%)	**Hybrid**	**0.0064**	**0.0088**	**0.0060**	**0.0069**
	LSTM	0.0183	0.0365	0.0292	0.0152
	RL	0.0100	0.0129	0.0108	0.0102
	BPINN-1^19^	0.3711	0.3146	0.3071	0.2789
	BPINN-2^19^	0.2685	0.2937	0.2268	0.2193
	FNN^19^	0.7734	1.0392	0.9856	0.7246
${R}^{2}$	**Hybrid**	**0.9958**	**0.9940**	**0.9945**	**0.9916**
	LSTM	0.9657	0.8966	0.8708	0.9587
	RL	0.9897	0.9871	0.9823	0.9812

Significant values are in bold.

These results clearly highlight the superior performance of our proposed approach compared to conventional methods. Additionally, BPINN-1 and BPINN-2, despite incorporating physics-informed structures, fail to capture battery behavior effectively, resulting in significantly higher errors. Furthermore, FNN performs the worst, demonstrating its inability to model the underlying dynamics. Additionally, the results suggest that while physics-informed ML approaches enhance interpretability compared to standard data-driven models, their predictive accuracy remains lower than that of our hybrid method. The two-stage design of our LSTM + PPO model enables better temporal representation learning and constraint enforcement, resulting in consistently lower error metrics and higher robustness across various datasets.

.The Hybrid model consistently outperforms the other methods in all metrics, highlighting its superior accuracy and generalization capability across different battery datasets. The proposed hybrid model’s consistent performance across all datasets underscores its robust generalization ability. It handles variations in battery behavior more effectively than either of the baseline models. The hybrid model’s consistent outperformance across all battery datasets can be attributed to its integration of data-driven sequence modeling and adaptive reinforcement learning correction. The LSTM component excels at capturing long-term temporal dependencies and nonlinear degradation trends in battery behavior, effectively learning patterns such as gradual SOH decline and transient anomalies.

## Evaluation on CALCE CS2 dataset

**Fig. 6:**
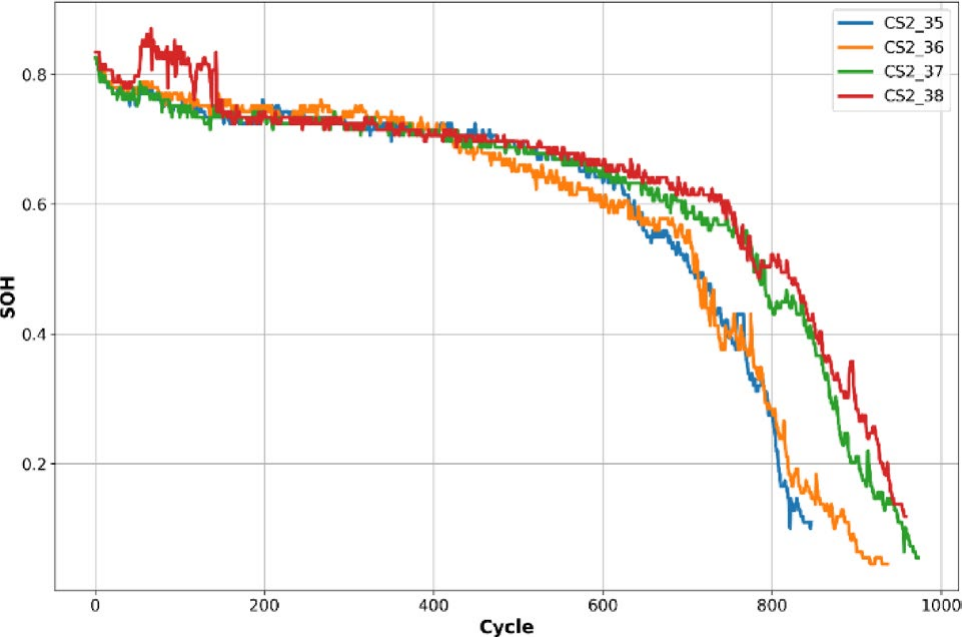
SOH of the CS2 dataset over battery life

To further evaluate the generalization and effectiveness of the proposed hybrid model, another dataset, CALCE CS2^9^, is included, which is provided by the CALCE Prognostics and Health Management (PHM) Group at the University of Maryland. All CS2 cells underwent the same charging profile, which was a standard constant current/constant voltage protocol with a constant current rate of 0.5 °C until the voltage reached 4.2V. Then 4.2V was sustained until the charging current dropped to below 0.05A. Four batteries CS2_35, CS2_36, CS2_37 and CS2_38 are used. The batteries are all cycled at a constant temperature of 1 °C^9^. These batteries offer a diverse and realistic benchmark for validating battery health prediction models. Each battery was subjected to controlled charge–discharge cycles under varying operational conditions, resembling real-world usage patterns. These cells followed a standard constant current-constant voltage (CC-CV) charging protocol, and degradation was monitored through key parameters: constant voltage charging time (CVCT), constant current charging time (CCCT), internal resistance, and cycle number. These features evolved as the battery ages, providing rich temporal features for predictive modeling, and were used to train our models. Unlike the NASA dataset, where SOH must often be computed or estimated from key features measurements, the CS2 dataset provides explicit cycle-wise SOH values shown in Fig. 6, making it ideal for benchmarking supervised learning models. The data for each battery (CS2_35 through CS2_38) contains 3712 complete cycles, with each row representing one full cycle. This granularity simplifies temporal modeling while allowing for precise validation of degradation patterns. Making it ideal to test the generalization capability of the proposed models after training on the NASA data, providing a robust evaluation across different chemistries, operating conditions, and real-world degradation scenarios.

The inclusion of these batteries in model evaluation is valuable because they present unique degradation behaviors and lifespan ranges, typically spanning several hundred cycles until end-of-life. The same architecture model was used, along with the same number of training epochs .

The Results consistently showed that the hybrid model achieved superior performance, with significantly lower MAE and RMSE and higher R^2^ scores across all battery cases. These results clearly demonstrate the superiority of the proposed Hybrid (LSTM + PPO) model over standalone LSTM and RL models across all evaluated battery datasets (CS2_35 to CS2_38). In terms of MAE, RMSE, and ${R}^{2}$ score.

**Fig. 7:**
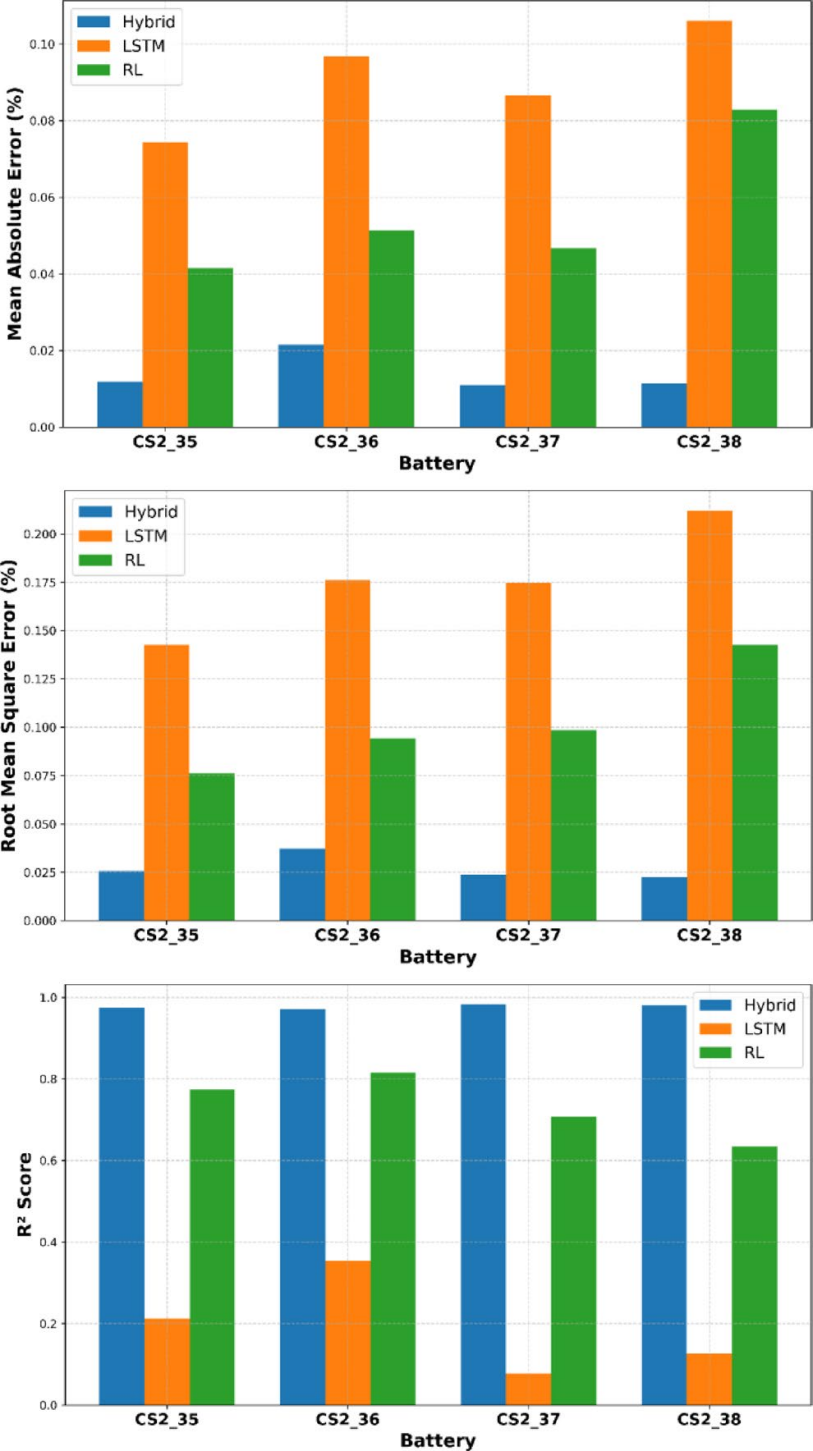
Evaluation Metrics Comparison for Different CALCE CS2 Batteries.

**Fig. 8:**
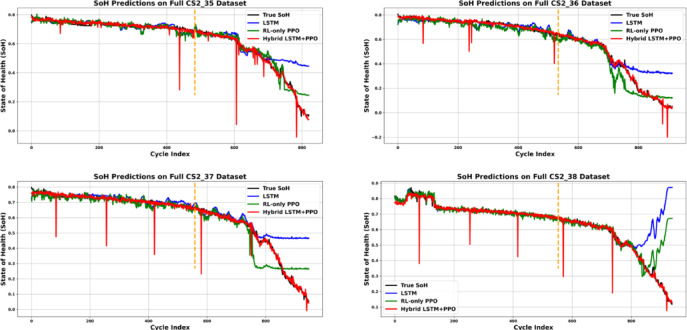
Evaluation Metrics Comparison for Different CALCE CS2 Batteries.

The Hybrid model consistently achieves the lowest values, indicating greater prediction accuracy and stability. Furthermore, the Hybrid model outperforms LSTM by a substantial margin. On CS2_35, the MAE drops from 0.0743 (LSTM) to 0.0118 (Hybrid) and the RMSE from 0.1426 to 0.0255, highlighting its ability to correct the systematic biases inherent in sequence modeling. Compared to standalone RL, the Hybrid approach also yields lower error metrics and significantly improved R^2^ scores, demonstrating better generalization and fit to the actual SOH degradation trends, as shown in the Table 4 and Fig. 7. Predictions for the whole life cycle of batteries are presented in Fig. 8.

**Table 4:** Evaluation METRICS of the CALCE CS2 Dataset Batteries.

Battery		CS2_35	CS2_36	CS2_37	CS2_38
MAE	Hybrid	**0.0118**	**0.0215**	**0.0110**	**0.0115**
	LSTM	0.0743	0.0968	0.0865	0.1060
	RL	0.0414	0.0514	0.0467	0.0828
RMSE	Hybrid	**0.0255**	**0.0374**	**0.0238**	**0.0224**
	LSTM	0.1426	0.1762	0.1746	0.2119
	RL	0.0763	0.0940	0.0985	0.1423
${R}^{2}$	Hybrid	**0.9749**	**0.9708**	**0.9828**	**0.9805**
	LSTM	0.2121	0.3533	0.0776	0.1254
	RL	0.7742	0.8159	0.7068	0.6335

Significant values are in bold.

These improvements confirm that combining the temporal learning capabilities of LSTM with the adaptive correction mechanism of reinforcement learning yields more robust and accurate predictions of battery health across diverse operating conditions.

## Conclusion

This work proposed a hybrid SOH prediction framework that combines LSTM neural networks with PPO-based reinforcement learning to address the challenges of accurately forecasting battery health over time. Initially validated on the NASA battery dataset, the hybrid model demonstrated clear improvements over standalone LSTM and RL approaches, as well as state-of-the-art methods. To further validate its robustness and generalizability, we applied the model to a different real-world dataset, the CS2 battery series (CS2_35 to CS2_38). Results consistently showed that the hybrid model achieved superior performance, with significantly lower MAE and RMSE and higher R2 scores across all battery cases. This confirms that integrating data-driven sequence modeling with adaptive correction mechanisms enables the hybrid approach to capture both the temporal degradation patterns and dynamic variations in battery behavior more effectively. These findings highlight the effectiveness and reliability of the proposed model in diverse operating conditions and datasets, making it a strong candidate for practical deployment in battery management systems.

## Data Availability

The study uses the following publicly available datasets: 1. NASA Battery Dataset—used for training, available via NASA’s Prognostics Center of Excellence repository: [https://www.nasa.gov/content/prognostics-center-of-excellence-data-set-repository] (https:/www.nasa.gov/content/prognostics-center-of-excellence-data-set-repository) 1. CALCE CS2 Battery Dataset (CS2_35 to CS2_38)—used for external validation and cross-testing: Available from the University of Maryland Center for Advanced Life Cycle Engineering (CALCE) battery data archive: https://calce.umd.edu/battery-data
